# Treatment Landscape of Renal Cell Carcinoma

**DOI:** 10.1007/s11864-023-01161-5

**Published:** 2023-12-28

**Authors:** Yu-Wei Chen, Luke Wang, Justine Panian, Sohail Dhanji, Ithaar Derweesh, Brent Rose, Aditya Bagrodia, Rana R. McKay

**Affiliations:** 1 Division of Hematology Oncology, University of California San Diego, San Diego, CA USA; 2 Department of Urology, University of California San Diego, San Diego, CA USA; 3 School of Medicine, University of California San Diego, San Diego, CA USA; 4 Department of Urology, The University of Tennessee Health Science Center, Memphis, TN USA; 5 Department of Radiation Oncology, University of California San Diego, San Diego, CA USA

**Keywords:** Renal cell carcinoma, Immunotherapy, Immune checkpoint inhibitor, Kidney cancer

## Abstract

The treatment landscape of renal cell carcinoma (RCC) has evolved significantly over the past three decades. Active surveillance and tumor ablation are alternatives to extirpative therapy in appropriately selected patients. Stereotactic body radiation therapy (SBRT) is an emerging noninvasive alternative to treat primary RCC tumors. The advent of immune checkpoint inhibitors (ICIs) has greatly improved the overall survival of advanced RCC, and now the ICI-based doublet (dual ICI-ICI doublet; or ICI in combination with a vascular endothelial growth factor tyrosine kinase inhibitor, ICI-TKI doublet) has become the standard frontline therapy. Based on unprecedented outcomes in the metastatic with ICIs, they are also being explored in the neoadjuvant and adjuvant setting for patients with high-risk disease. Adjuvant pembrolizumab has proven efficacy to reduce the risk of RCC recurrence after nephrectomy. Historically considered a radioresistant tumor, SBRT occupies an expanding role to treat RCC with oligometastasis or oligoprogression in combination with systemic therapy. Furthermore, SBRT is being investigated in combination with ICI-doublet in the advanced disease setting. Lastly, given the treatment paradigm is shifting to adopt ICIs at earlier disease course, the prospective studies guiding treatment sequencing in the post-ICI setting is maturing. The effort is ongoing in search of predictive biomarkers to guide optimal treatment option in RCC.

## Introduction

Histologically, clear cell renal cell carcinoma (ccRCC) represents approximately 75–80% of RCC and the remainder of tumors classified as non-clear cell RCC (nccRCC). There have been tremendous advancements in understanding the biology of both ccRCC and nccRCC in the past three decades which has led to improved therapeutic options and prolonged survival. Despite this progress, it is estimated that there will be 81,800 new RCC cases diagnosed in the USA in 2023 and an estimated 14,890 deaths from RCC [1], suggesting an continued unmet need to improve outcomes in this disease. The current review aims to discuss the treatment modalities for localized and advanced RCC with a focus on assessing the landmark prospective studies.

## Treatment of localized disease

The majority of patients with RCC are diagnosed with localized tumors. For patients with localized disease, the standard of care remains surgical excision, either through radical or partial nephrectomy. Active surveillance with or without tumor biopsy or tumor ablation are also options in appropriately selected patients. Despite definitive local therapy, a subgroup of patients will develop recurrent or advanced RCC with an estimated 5-year recurrence free survival ranged from 42 to 98% [2].

### Risk stratification for localized disease

Risk stratification tools for localized disease have been developed over the years to identify patients with an increased likelihood of cancer recurrence and adverse outcomes. For localized RCC, tumor stage and nodal disease status in the TNM staging system are important prognostic factors. The tumor stage considers tumor size and disease extent. Nodal disease is infrequent but when present is invariably associated with poor prognosis [3]. In addition to the TNM staging, higher tumor grade is associated with increased disease recurrence independent of tumor stage [4]. Many nomograms have been developed combining the TNM staging system with additional clinical and pathologic factors (e.g., tumor grade, histologic features, performance status, tumor necrosis, clinical symptoms, and margin status) to aid in prognostication in the localized disease [5]. Currently, there is no validated biomarker beyond traditional clinical characteristics and histopathology in localized RCC.

### Surgical management

Radical nephrectomy (RN) was historically served as the standard for management of all localized renal masses. However, partial nephrectomy (PN), using both open and robotic approaches, has now been recognized as the surgical standard for management of T1 (≤7 cm) renal masses. In T2 renal masses (>7 cm and limited to the kidney), PN is an acceptable approach in selected patients with the potential to preserve renal functional [6, 7]. In the setting of T3 disease, characterized by tumor extension beyond the renal cortex, RN has been viewed as the standard for definitive surgical management. However, recent literatures have examined the role of PN in this setting which showed acceptable outcomes in selected patients for which nephron-sparing surgery would be indicated [7–9].

### Ablative approaches

Radiofrequency ablation (RFA) and cryoablation (CA) have been developed in recent years for selected patients with small renal masses [7]. Common indications include patients who are poor surgical candidates or who prefer a non-surgical approach. Current literature supports these ablative techniques as potential alternatives for patients with T1a masses (≤3 cm). A renal mass biopsy is recommended prior to or concomitant with ablation [10] to confirm histology and guide surveillance. Recent systematic review showed that ablation exhibited increased risk of local recurrence (5-year recurrent free rate: 94%) though this may be managed with repeat ablations with similar outcomes when treated with surgery [7, 11, 12].

### Active surveillance

Increasing knowledge regarding the natural history of small renal masses, particularly in the context of competing patient comorbidities, led to interest in active surveillance as an initial management strategy for patients with small renal masses. Current guidelines endorse active surveillance as a part of the shared-decision making process in patients with small renal masses [13].

## Adjuvant therapy

Cytokine therapy, vascular endothelial growth factor (VEGF) tyrosine kinase inhibitor (TKI), and mammalian target of rapamycin (mTOR) inhibitors were investigated in the adjuvant setting with mixed results [5, 14]. Sunitinib is the only FDA-approved VEGF TKI in resected high-risk ccRCC based on the positive phase III S-TRAC trial [15]. The primary endpoint was met which showed an improved disease-free survival (DFS) (6.8 years vs 5.6 years in placebo; *p*-value: 0.03). However, several other adjuvant studies (ASSURE, SORCE, PROTECT, ATLAS) [16–19] failed to demonstrate a clinical benefit of VEGF TKIs. Given the considerable toxicities and lack of proven overall survival (OS) benefit, adjuvant sunitinib has limited use in clinical practice.

Given the success of immune checkpoint inhibitor (ICIs) in the advanced disease setting, the efficacy of ICIs in the adjuvant/perioperative setting has also been evaluated. The KEYNOTE 564 was the first and the only positive adjuvant trial of ICIs in RCC. The study investigated 12-month pembrolizumab in patients with intermediate-high risk (pT2N0M0 grade 4 or sarcomatoid, or pT3N0M0 any grade) or high-risk (pT4N0M0 any grade, or pTxN1M0 any grade) ccRCC after definitive radical or partial nephrectomy [20••]. Patients with M1 disease rendered no evidence of disease (NED) ≤1 year from surgery were also included (brain or bone metastasis not eligible). At extended median follow-up of 30 months, adjuvant pembrolizumab continued to show improved DFS compared to placebo (78.3% vs 67.3% at 24 months, HR 0.63, 95% CI 0.50-0.80; *p* < 0.0001) [21••]. The OS data has not matured yet although the HR continued trending down. Of note, pembrolizumab generally has a favorable tolerability by patients but the toxicities are not negligible (20.7% patients in the pembrolizumab arm required treatment discontinuation due to adverse events and 7.4% patients required high-dose glucocorticoids). The judgement to offer adjuvant pembrolizumab should be based on individual’s disease recurrent risk and consider risk-benefit ratio through shared decision-making.

The promising results of KEYNOTE 564 was accompanied by a series of parallel negative adjuvant trials investigating other ICIs [5] (Table 1). The trials differed with regard to eligibility criteria, treatment, and design. These negative studies raised debates regarding whether the efficacy of ICIs exist in the adjuvant setting for RCC. There are many nuances that may explain the negative findings which are beyond the scope of this review [5]. In addition, cross-trial comparison is always challenging. Currently, adjuvant pembrolizumab is the only FDA-approved ICI in resected ccRCC. Highly sensitive biomarkers of minimal residual disease would identify patients most likely to benefit from adjuvant therapy while sparing those cured with surgery alone from unnecessary treatment-related toxicity with no oncologic benefit.

**Table 1:** Adjuvant immune checkpoint trials for localized or locally advanced renal cell carcinoma

Trial	Treatment arms	Treatment length	Histology	M1 NED eligibility	Primary endpoint	Hazard ratio 95% CI
IMmotion010	Atezolimumab vs placebo	1 year	Clear cell component or sarcomatoid	Yes	DFS	0.93 (95% CI 0.75–1.15)
CheckMate 914	Nivolumab + Ipilumumab (part A) vs Nivolumab (part B) vs. Placebo	6 months	Clear cell predominant	No	DFS	0.92 (95% CI 0.71–1.19)
PROSPER RCC	Neoadjuvant Nivolumab + Adjuvant Nivolumab vs. Surgery + surveillance	10 months (1 month neoadjuvant; 9 months adjuvant)	Any malignant RCC	Yes	RFS	0.97 (95% CI 0.74–1.28)
Keynote 564	Pembrolizumab vs. Placebo	1 year	Clear cell component	Yes	DFS	0.68 (95% CI 0.53–0.87)
RAMPART	Durvalumab vs Durvalumab + tremelimumab vs observation	1 year	Any RCC (except for collecting duct, medullary, or transitional)	No	DFS	Pending
Litespark-022	Belzutifan + pembrolizumab vs placebo + pembrolizumab	1 year	Clear cell component	Yes	DFS	Pending

*Abbreviations*: *vs* versus, *DFS* disease-free survival, *RFS* recurrence-free survival, *NED* no evidence of disease, *G1-4* grade 1 to 4, *CI* confidence interval

## Neoadjuvant therapy

Neoadjuvant therapy with TKIs prior to nephrectomy has been shown to downstage the RCC tumors with high-risk features (e.g., large unresectable kidney mass, tumor thrombus invading the inferior vena cava) although currently there is no approved standard neoadjuvant therapy in RCC [22]. Given the success of ICI in the advanced disease setting, it has been hypothesized that neoadjuvant ICI could elicit robust immune response with the intact primary tumor providing high antigen load [23]. Two small studies investigating ICI monotherapy have showed safety/feasibility of neoadjuvant approach in RCC; however, radiographic response rate was low [24, 25]. There are several ongoing neoadjuvant RCC trials investigating ICI-based combinations [26].

## Treatment of advanced or metastatic disease

### Risk stratification for advanced disease

The Memorial Sloan Kettering Cancer Center (MSKCC) model was developed in the cytokine era and identified key prognostic factors [27] that are still pertinent to contemporary patients. The current widely used International Metastatic Renal Cell Carcinoma Database Consortium (IMDC) model was initially established in the targeted therapy era [28••] and is composed of two clinical factors (Karnofsky performance status/time from original RCC diagnosis to initiation of systemic therapy) and four laboratory factors from blood tests (hemoglobin/neutrophil/platelet/calcium). The IMDC model categorizes patients into favorable risk (0 factor), intermediate risk (1–2 factors), or poor risk (≥3 factors) based on the number of adverse prognostic factors and the risk groups are associated with differential OS. It was initially validated for patients with ccRCC and has subsequently been able to reliability predict survival for patients with nccRCC [29]. While initially developed as a prognostic model for patients initiating targeted therapy, the IMDC risk has been incorporated into landmark studies of immunotherapy combination regimens for baseline risk stratification. It has maintained relevance in the immunotherapy era given that front-line studies were specifically designed with endpoint in select IMDC risk-groups.

### Frontline treatment for metastatic renal cell carcinoma (mccRCC)

The treatment paradigm for mccRCC has advanced remarkably in the past three decades. Up until 2005, treatment options for patients with mRCC were largely limited to cytokine-based therapies including interferon and interleukin-2. Given the advancements in our understanding of RCC pathogenesis, VEGF targeting agents entered the clinic in 2005 and remained the standard frontline treatment options until 2018. Currently, immune checkpoint inhibitor (ICI)–based doublets have significantly improved the OS of mccRCC and are now the new standard of care. There are two categories of ICI-based doublets: (1) the dual ICI combination targeting cytotoxic T lymphocyte-associated antigen 4 (CTLA-4) and programmed cell death 1 (PD-1) protein (ICI-ICI combination) and (2) the combination of an anti-PD1 ICI plus a vascular endothelial growth factor (VEGF) tyrosine kinase inhibitor (ICI-TKI combination). There are four ICI-based doublets that have demonstrated OS benefit compared to sunitinib in the intention-to-treatment population (ITT) (Table 2): ipilimumab/nivolumab (ICI-ICI combination) [30], pembrolizumab/axitinib (ICI-TKI combination) [31••], nivolumab/cabozantinib (ICI-TKI combination) [32••], and pembrolizumab/lenvatinib (ICI-TKI combination) [33••].

**Table 2:** Efficacy of frontline ICI-based doublets in mccRCC

	ICI-ICI	IO-TKI		
	CheckMate 214 Ipilimumab/Nivolumab	KEYNOTE 426 Pembrolizuamb/Axitinib	CheckMate 9ER Nivolumab/Cabozantinib	CLEAR Pembrolizumab/Lenvatinib
Comparator: sunitinib				
Median follow-up (months)	67.7 (minimum: 60)	67.2 (minimum: 60)	44 (minimum: 36.5)	48
Intention-to-treat				
mOS HR (95% CI)	55.7 vs 38.4 0.72 (0.62–0.85)	47.2 vs 40.8 0.84 (0.71–0.99)	49.5 vs 35.5 0.70 (0.56–0.87)	53.7 vs 54.3 0.79 (0.63–0.99)
mPFS HR (95% CI)	12.3 vs 12.3 0.86 (0.73–1.01)	15.7 vs 11.1 0.69 (0.59–0.81)	16.6 vs 8.4 0.59 (0.49–0.71)	23.9 vs 9.2 0.47 (0.38–0.57)
ORR (%)	39 vs 32	61 vs 40	56 vs 28	71 vs 37
CR (%)	12 vs 3	11.6 vs 4.0	13 vs 5	18.3 vs 4.2
Median duration of response (months)	NR vs 24.8	23.6 vs 15.3	22.1 vs 16.1	26.7 vs 14.7
Primary PD (%)	17.6	11.6	7	5.4
Intermediate/poor risk				
mOS HR (95% CI)	47 vs 26.6 0.68 (0.58–0.81)	42.2 vs 29.3 0.76 (0.62–0.93)	49.5 vs 29.2 0.65 (0.51–0.83)	47.9 vs 34.3 0.74 (0.57–0.96)
mPFS HR (95% CI)	11.6 vs 8.3 0.73 (0.61–0.87)	13.8 vs 8.3 0.68 (0.56–0.82)	15.6 vs 7.1 0.56 (0.46–0.69)	22.1 vs 5.9 0.43 (0.34–0.55)
ORR (%)	42 vs 27	59 vs 35	53 vs 23	72 vs 29
Favorable risk				
mOS HR (95% CI)	74.1 vs 68.4 0.94 (0.65–1.37)	60.3 vs 62.4 1.10 (0.79–1.54)	NR vs 47.6 1.07 (0.63–1.79)	NR vs 59.9 0.94 (0.58–1.52)
mPFS HR (95% CI)	12.4 vs 28.9 1.60 (1.13–2.26)	20.7 vs 17.9 0.76 (0.57–1.02)	21.4 vs 13.9 0.72 (0.49–1.05)	28.6 vs 12.9 0.50 (0.35–0.71)
ORR (%)	30 vs 52	69 vs 50	68 vs 46	68 vs 50

*mOS* median overall survival, in months ; *mPFS* median progression-free survival, in months; *HR (95%CI)* hazard ratio (95% confidence interval); *NR* not reached

While these IO combination regimens are life prolonging, the two categories have distinct efficacy profile. One distinguishable feature of the ICI-ICI combination of ipilimumab/nivolumab is the long-term durable response: after a minimum follow-up of 5 years (median: 67.7 months), the duration of response (DOR) of ipilimumab/nivolumab had continued not been reached (NR) [34••]. For the ICI-TKI combinations, the median DOR ranged from 22 to 26.7 months (median follow-up time: 48–67.2 months) [35••, 36••, 37••]. On the contrary, the ICI-TKI combinations generally have longer PFS (median PFS range: 15.7–23.9 months vs 12.3 months in ipilimumab/nivolumab) and higher objective response rate (ORR) (range: 56–71% vs 39% in ipilimumab/nivolumab) [35••, 36••, 37••]. The primary disease progression (PD) rate also favors ICI-TKI combinations (range: 5.4–11.6% vs 17.6% in ipilimumab/nivolumab) [35••, 36••, 37••]. Lastly, given the potential persistent efficacy after ICI discontinuation, treatment-free survival (TFS), which was defined by the area between two Kaplan-Meier curves (time to protocol therapy cessation and time to subsequent systemic therapy), was investigated in the CheckMate 214. At 42-month follow-up, mean TFS was more than doubled in ipilimumab/nivolumab vs sunitinib (6.9 vs 3.1 months) in IMDC intermediate/poor risk and tripled in IMDC favorable risk (11.0 vs 3.7 months). Mean TFS with ≥grade 3 adverse events for ipilimumab/nivolumab were minor (0.6 months in intermediate/poor risk and 0.9 months in favorable risk). This analysis suggests that ipilimumab/nivolumab not only prolongs OS but patients also spend more time off therapy without toxicities with maintained disease control.

Currently, there is no level one evidence to suggest superiority of one combination over another and there are caveats in cross-trial comparisons. Decision making is empirically based on the treating physician’s interpretation of the existing data and considerations of burden of disease, toxicities, and patient fitness. Overall, the clinical efficacy of ipilimumab/nivolumab is mainly driven by the IMDC intermediate and poor risk patients, while the ICI-TKI combinations generally show efficacy across the three IMDC risk categories (Table 2).

### IMDC intermediate/poor risk

- ICI-ICI combination

The Food and Drug Administration (FDA) approved ipilimumab/nivolumab as a frontline therapy in the IMDC intermediate and poor risk patients based on the Checkmate 214 study [30]. The Co-primary endpoints (ORR/PFS/OS) were investigated among the IMDC intermediate and poor risk patients comparing ipilimumab/nivolumab versus sunitinib. After a minimum of 5-year follow-up [34••], the superior efficacy of ipilimumab/nivolumab were maintained (ORR: 42% vs 27%, PFS: 11.6 months vs 8.3 months, OS: 47 months vs 26.6 months) (Table 2). One recognizable hallmark of the dual ICI-ICI combination was the durable long-term efficacy as demonstrated at the tail of the PFS curve: the PFS had plateaued at 24 months (PFS at 24 months: 36.4%; PFS at 60 months: 31%) indicating that a subset of the patients had not progressed since randomization. In addition, the median DOR had not been reached (vs 19.7 months in sunitinib) [30].

- ICI-TKI combination

The KEYNOTE 426 (pembrolizumab/axitinib) [31••], CheckMate 9ER (nivolumab/cabozantinib) [32••], and CLEAR (pembrolizumab/lenvatinib) [33••] investigated an anti-PD1 ICI plus an VEGF TKI in frontline mccRCC. The three ICI-TKI combinations all demonstrated OS benefit over sunitinib in the ITT population and received FDA approvals irrespective of IMDC risk. Subgroup analyses of the three ICI-TKI combinations all demonstrated superior ORR, PFS, and OS over sunitinib in the IMDC intermediate and poor risk and the efficacy were maintained after extended follow-up (Table 2) [35••, 36••, 37••].

### IMDC favorable risk

- ICI-ICI combination

Given the primary endpoint of CheckMate 214 was designed specifically in the intermediate-poor risk patients, the study was underpowered to assess statistically significant differences in OS in the favorable risk group (*n*). At 67.7 months of follow-up, ipilimumab/nivolumab demonstrated similar OS to sunitinib (median OS: 74.1 months vs 68.4 months, HR: 0.94, 95% CI: 0.65–1.37), shorter PFS (median PFS: 12.4 months vs 28.9 months, HR: 1.60, 95% CI: 1.13–2.26), and lower ORR (30% vs 52%) compared to sunitinib monotherapy [30]. Although the OS benefit was similar, the OS hazard ratios of ipilimumab/nivolumab had trended down over time (from 1.45 to 0.94; Table 3). Prolonged follow-up time would be required to formally investigate an OS benefit in this subgroup given the relatively indolent course of favorable risk disease and the OS would also be impacted by subsequent therapies. However, there was clearly a subset of favorable risk patients that benefited from ipilimumab/nivolumab as the median DOR was nearly doubled (61.5 months vs 33.2 months) among responders and the complete response (CR) rate was higher (13% vs 6%). Clinically, it is not unreasonable to consider ipilimumab/nivolumab in selected favorable risk patients (e.g., low volume disease) if the treatment goal is to prioritize long-term durable efficacy. Future studies are needed to identify biomarkers to select the ideal IMDC favorable risk patients to receive frontline ipilimumab/nivolumab combination.

- ICI-TKI combination

**Table 3:** OS hazard ratios at extended follow-up by IMDC risk

	CheckMate 214 Ipilimumab/Nivolumab	KEYNOTE 426 Pembrolizuamb/Axitinib	CheckMate 9ER Nivolumab/Cabozantinib	CLEAR Pembrolizumab/Lenvatinib
Intermediate/poor risk				
(Follow-up time)	(25.2 months)	(12.8 months)	(18.1 months)	(26.6 months)
		Intermediate	Intermediate	Intermediate
	0.63 (99.8% CI: 0.44–0.89)	0.53 (95% CI: 0.35–0.82)	0.70 (95% CI: 0.46–1.07)	0.66 (95% CI: 0.47–0.94)
		Poor	Poor	Poor
		0.43 (95% CI: 0.23–0.81)	0.37 (95% CI: 0.21–0.66)	0.50 (95% CI: 0.23–1.08)
	(32.4 months)	(30.6 months)	(32.9 months)	(48 months)
			Intermediate	
	0.66 (95% CI: 0.54–0.80)	0.63 (95% CI: 0.50–0.81)	0.74 (95% CI: 0.54–1.01)	0.74 (95% CI: 0.57–0.96)
			Poor	
			0.49 (95% CI: 0.31–0.79)	
	(43.6 months)	(42.8 months)	(44 months)	
	0.66 (95% CI: 0.55-0.80)	0.64 (95% CI: 0.52–0.80)	0.65 (0.51–0.83)	
	(55 months)	(67 months)		
	0.65 (95% CI: 0.54–0.78)	0.76 (95% CI: 0.62–0.93)		
	(67.7 months)			
	0.68 (95% CI: 0.58–0.81)			
Favorable risk				
(Follow-up time)	(25.2 months)	(12.8 month)	(18.1 months)	(26.6 months)
	1.45 (99.8% CI: 0.51–4.12)	0.64 (95% CI: 0.24–1.68)	0.84 (95% CI: 0.35–1.97)	0.86 (95% CI: 0.38–1.92)
	(32.4 months)	(30.6 months)	(32.9 months)	(48 months)
	1.22 (95% CI: 0.73–2.04)	1.06 (95% CI: 0.60–1.86)	1.03 (95% CI: 0.55–1.92)	0.94 (95% CI: 0.58–1.52)
	(43.6 months)	(42.8 months)	(44 months)	
	1.19 (95% CI: 0.77–1.85)	1.17 (95% CI: 0.76–1.80)	1.07 (95% CI: 0.63–1.79)	
	(55 months)	(67 months)		
	0.93 (95% CI: 0.62–1.40)	1.10 (95% CI: 0.79–1.54)		
	(67.7 months)			
	0.94 (95% CI: 0.65–1.37)			

Despite the higher ORR and numerically longer PFS with the ICI-TKI combinations over sunitinib (Table 2), whether the OS benefit of IO-TKI combinations also exists in the IMDC favorable risk subgroup has engendered debates. The disputes were due to the subgroup analyses of the three ICI-TKI trials (KEYNOTE-426, CheckMate 9ER, CLEAR) did not show a clear OS benefit over sunitinib after extended follow-up (Table 3). Of note, the landmark trials were not statistically powered to investigate the ICI-TKI combinations specifically in the IMDC favorable risk and the subgroup analyses should be interpreted as hypothesis-generating. In general, the efficacy of the three ICI-TKI doublets is considered superior to sunitinib and the NCCN guideline lists them as category 1 recommendation (but not sunitinib) for IMDC favorable risk disease [38]. Attention must be paid to the toxicities associated with the ICI-TKI doublets given the indolent biology of IMDC favorable disease and patients may stay on therapy for prolonged duration. The results from the subgroup analyses have also led to debates whether sequential approaches of systemic therapy (as opposed to ICI-doublets) in the IMDC favorable risk should be considered and this hypothesis can only be verified in prospective trials.

### Triplet regimen: ICI-ICI-TKI

With the success of ICI-ICI and ICI-TKI in the frontline setting of mccRCC, it is rational to hypothesize that combining VEGF and dual ICI-ICI treatments would improve outcomes. COSMIC-313 was a phase III randomized placebo-controlled trial evaluating the triplet of ipilimumab/nivolumab/cabozantinib (40 mg daily) (ICI-ICI-TKI) vs ipilimumab/nivolumab (ICI-ICI) in the IMDC intermediate and poor risk patients [39••]. The primary endpoint of PFS was assessed in the first enrolled 550 patients, and the triplet regimen showed an improved PFS over ipilimumab/nivolumab (mPFS: NR vs 11.3; PFS at 12 months: 57% vs 49%, HR: 0.73, 95% CI: 0.57–0.94, *p* = 0.01). The ORR was numerically higher with the triplet: 43% (95% CI: 37–49%) vs 36% (95% CI: 30–42%). The OS data has not matured. When stratified by IMDC risk, ipilimumab/nivolumab/cabozantinib improved the PFS in the intermediate subgroup (HR: 0.63, 95% CI: 0.47–0.85) but interestingly not in the poor risk (HR: 1.04, 95% CI:0.65–1.69). In terms of toxicity profile, the triplet arm had higher grade 3/4 adverse events (79% vs 56%), more patients required dose holds of any medication (90% vs 70%), more patients required dose reduction of cabozantinib (54% vs 20%; average daily dose: 23.2 mg vs 36.1 mg of placebo), and less patients finished all four doses of ipilimumab (58% vs 73%). It is reasonable to postulate that the less than expected efficacy of this triplet regimen is the results of inadequate drug delivery due to significant toxicities.

Of note, the patient population in the COSMIC-313 was different from the aforementioned ICI-doublet trials. In addition to not including IMDC favorable risk disease, there were less patients who had radical nephrectomy (64% vs 74-83%) and less tumors with sarcomatoid features (6.3% vs 8–18%) [40].

Therefore, it is challenging to make comparisons across trials. Although not practice-changing, the COSMIC-313 should be accoladed as the first phase III trial that used a contemporary standard-of-care control (ipilimumab/nivolumab) and demonstrated the feasibility of triplet regimen in the frontline setting.

There is ongoing phase III study investigating the triplet regimen of pembrolizumab/belzutifan (HIF inhibitor)/lenvatinib or pembrolizumab/quavonlimab(anti-CTLA-4 antibody)/lenvatinib vs pembrolizumab/lenvatinib [41•]. Another phase III trial using an adaptive design (PDIGREE) that investigates maintenance nivolumab/cabozantinib vs nivolumab after ipilimumab/nivolumab induction among non-CR and non-PD patients and the results will provide additional information in the field [42•].

### ICI monotherapy

Although not preferred, anti-PD-1 ICIs (pembrolizumab, nivolumab) have demonstrated anti-tumor activity as a single agent. The KEYNOTE 427 cohort A investigated pembrolizumab monotherapy in advanced ccRCC (*N* = 110) [43]. The ORR was 36% (95% CI: 27–46), disease control rate was 58% (95% CI: 45–68), and the median DOR was 19 months. The efficacy of nivolumab monotherapy in ccRCC was demonstrated in the HCRN GU 16-260 Part A (*N* = 123) [44]. The ORR was 34% (95% CI: 26–43), and the median DOR was 27.6 months. For patients who are less fit for ICI combinations, it is not unreasonable to offer ICI monotherapy to avoid additional toxicities.

### Biomarker-driven trials in RCC

Tremendous efforts have been made to identify predictive biomarkers in mRCC to guide treatment selection [45]. BIONIKK was the first trial in mRCC which used gene expression signatures established in the TKI era and proved feasibility of such biomark-driven approach in prospective trial [46]. Currently, there is no available biomarker to guide decision between an ICI-ICI vs ICI-TKI combination in mRCC. A correlative study of the phase III IMmotion 151 used RNA-sequencing and categorized RCC tumors into seven biologically distinct clusters which had differential response to ICI [47]. An ongoing phase II OPTIC RCC trial adopts a biomarker-driven design and uses those clusters as predictive biomarker to assign protocol treatment between an ICI-TKI combination (nivolumab/cabozantinib) or an ICI-ICI combination (ipilimumab/nivolumab)[48]. RNA-sequencing will be performed on baseline tumor tissue to predict tumor cluster. The hypothesis is that the efficacy of the given ICI-doublet will be enhanced in biomarker selected patients compared to unselected patients in historical landmark trials.

## Treatment options for refractory mccRCC

While ICI-based doublets have greatly improved OS in mccRCC, there is a subset of patients that exhibit primary resistance to ICI-doublets (PD rate: 5–18%) [30, 31••, 32••, 33••] and the majority of patients eventually progress despite initial response (mPFS : 12.3–23.9 months) [30, 31••, 32••, 33••]. Treatment options for subsequent therapies depends on the received first-line treatment, and the current evidence guiding treatment sequencing after frontline ICI-based combinations continues to evolve.

### VEGF TKIs

Historical trials of VEGF TKIs (cabozantinib, axitinib, lenvatinib plus everolimus) [49–51] had demonstrated activities in the refractory setting after progression of prior TKIs although those trials included negligible patients who had prior ICIs. Recent studies of axitinib (NCT02579811) [52], cabozantinib (BREAKPOINT, CaboPoint) [53], and tivozanib (TIVO-3) [54••] either required progression after ICI therapy as trial eligibility or included a higher proportion of post-ICI patients. The prospective trials of VEGF TKIs in the refractory setting are summarized in Table 4.

- Axitinib

**Table 4:** Trials of VEGF TKIs for refractory mRCC

	Trial	Study design	Prior therapies	OS	PFS	ORR
Cabozantinib	METEOR	Phase III, Cabozantinib (*N* = 330) vs Everolimus (*N* = 328)	1L+ VEGF TKI (5% of cabozantinib arm and 4 % of everolimus arm received nivolumab)	21.4 vs 16.5 months HR: 0.66 95% CI: 0.53–0.83	7.4 vs 3.9 months HR: 0.51 95% CI: 0.41–0.62	17% vs 3%
	BREAKPOINT	Phase II single arm (*N* = 31)	Adjuvant or first line ICI	13.8 months 95% CI: 7.7–29.0	8.3 months 95% CI: 3.9–17.4	38% 95% CI: 20.7–57.7
	CaboPoint	Phase II, non-randomized Cohort A: post ICI-ICI (*N* = 125) Cohort B: post ICI-TKI (*N* = 125)	After 1L ICI-based combinations			Cohort A (*N* = 60)* 31.7% 95% CI: 20.3–45 Cohort B (*N* = 28)* 25% 95% CI: 10.7–44.9 *Interim analysis
	CANTANA (Control arm)	Phase II, randomized-controlled Trial Cabozantinib arm (*N* = 223)	62% had prior ICI; 29% had prior ipilimumab/nivolumab; 79% had prior VEGF TKI	24.8 months	9.3 months	28% (PD: 8%)
	Contact-3 (Control arm)	Phase II, randomized-controlled Trial Cabozantinib arm (*N* = 254)	100% had prior ICI 63% had prior VEGF TKIs	NE 95% CI: 21.1–NE	10.8 months	41% 95% CI: 35–47 (PD: 5%)
Axitinib	AXIS	Phase III Axitinib (*N* = 355) vs Sorafenib (*N* = 359)	1L+ systemic therapy (35% had cytokines; 8% had bevacizumab plus interferon alfa)	20.1 vs 19.2 months HR: 0.97 95% CI: 0.80–1.17	8.3 vs 5.7 months HR: 0.66 95% CI: 0.55–0.78	23% vs 12%
	NCT02579811	Phase II single arm (*N* = 40)	1L+ systemic therapy (63% had nivolumab monotherapy; 15% had ipilimumab/nivolumab)		8.8 months 95% CI: 5.7–16.6	45%
Tivozanib	Tivo-3	Phase III Tivozanib (*N* = 175) vs sorafenib (*N* = 175)	2 or 3 systemic therapies (1+ VEGF TKI); (27% of tivozanib arm and 25% of sorafenib arm received prior ICI and TKI)	ITT population 16.4 vs 19.2 months HR: 0.97 95% CI 0.75–1.24 ICI-treated subgroup HR: 0.84 95% CI: 0.50–1.40	ITT population 5.6 vs 3.9 months HR 0.73 95% CI: 0.56–0.94 ICI-treated subgroup 7.3 vs 5.1 months HR: 0.55 95% CI: 0.32–0.94	18% vs 8%
Lenvatinib plus everolimus	NCT01136733	Phase II, randomized lenvatinb plus everolimus (*N* = 50) vs everolimus (*N* = 50)	1L+ VEGF TKI	25.5 vs 15.4 months HR: 0.51 95% CI: 0.30–0.88	14.6 vs 5.5 months HR: 0.40 95% CI: 0.24–0.68	43% vs 6%

*NE* not evaluable, *PD* progression of disease

The efficacy of axitinib in the second-line setting was established in the AXIS trial and the results demonstrated a PFS benefit over sorafenib [50, 55]. A single-arm phase II study (NCT02579811) investigated individualized dosing of axitinib in previously treated mccRCC with ICI being the most recent therapy (*N* = 40; 63% had nivolumab monotherapy; 15% had ipilimumab/nivolumab) [52]. The result showed a median PFS of 8.8 months which did not meet the prespecified PFS threshold (9.5 months), and the ORR was 45%. A post hoc analysis among patients who discontinued ICI due to disease progression (*N* = 37) showed a median PFS of 9.2 months (95% CI: 6.2–16.6).

- Cabozantinib

In the phase III METEOR trial, cabozantinib demonstrated OS (median OS: 21.4 vs 16.5 months) and PFS (median PFS: 7.4 vs 3.9 months) benefit over everolimus after progression of prior VEGF TKIs [49] although only ≤5% patients had prior ICI treatment in this dataset [49].

Cabozantinib (60 mg daily) was evaluated in the phase II single-arm BREAKPOINT study which included contemporary patients progressed after adjuvant or first-line ICI [53]. Thirty patients were included for analysis (19 had ipilimumab/nivolumab, 7 had pembrolizumab/lenvatinib). The median PFS was 8.3 months (95% CI: 3.9–17.4) which met the prespecified threshold (mPFS: 7.4 months). Another on-going phase II trial, CaboPoint, is evaluating cabozantinib (60 mg daily) after frontline ipilimumab/nivolumab (cohort A, recruitment goal: *N* = 125) or ICI-TKI (cohort B, recruitment goal: *N* = 125) [56•]. An interim analysis was reported in February 2023: the ORR was 31.7% (95% CI: 20.3–45.0) and 25% (95% CI: 10.7–44.9) in cohort A (*N* = 60) and B (*N* = 28), respectively [57•].

The efficacy of cabozantinib in the post-ICI setting was also elucidated in two more recent randomized studies which used cabozantinib monotherapy as the control arm. In the CANTANA study [58] (62% had prior ICI; 29% had prior ipilimumab/nivolumab), the cabozantinib arm (*N* = 223) showed a median PFS of 9.3 months, ORR of 28%, and primary PD rate of 8%. In the Contact-3 study [59••] (100% had prior ICI), cabozantinib monotherapy (*N* = 254) showed a median PFS: 10.8 months, ORR of 41%, and primary PD rate of 5%.

- Lenvatinib plus everolimus

A phase II three-arm study randomized mccRCC patients who previously treated with VEGF TKIs to lenvatinib (18 mg daily) plus everolimus (5 mg daily), lenvatinib monotherapy (24 mg daily), or everolimus monotherapy (10 mg/daily) [51]. Lenvatinib plus everolimus met primary endpoint (PFS) over everolimus (median PFS: 14.6 vs 5.5 months, HR: 0.40, 95% CI: 0.24–0.68), and OS was improved in the updated post hoc analysis (median OS: 25.5 vs 15.4 months, HR: 0.51, 95% CI: 0.30–0.88). Lenvatinib monotherapy also demonstrated activity with improved PFS over everolimus (median PFS: 7.4 vs 5.5 months, HR: 0.61, 95% CI: 0.38–0.98) and numerically longer OS (median OS: 19.1 vs 15.4 months, HR: 0.68, 95% CI: 0.41–1.14). Of note, only 5 patients included in this trial (*N* = 153) had prior ICI therapy.

- Tivozanib

Tivo-3 is a phase III randomized trial which investigated tivozanib vs sorafenib in previously treated mccRCC (two or three previous systemic therapy; at least one VEGF TKI) [54••]. Patients (350) were included in this trial, and 26% had prior ICI-TKI combination. In the ITT population, tivozanib showed a PFS benefit over sorafenib (mPFS: 5.6 vs 3.9 months, HR: 0.73, 95% CI: 0.56–0.94) and met the trials primary endpoint. There was no OS difference (mOS: 16.4 vs 19.2 months, HR: 0.97, 95% CI: 0.75–1.24). The ORR was higher with tivozanib (18% vs 8%). In the ICI-treated subgroup, tivozanib showed improved PFS (mPFS: 7.3 vs 5.1 months, HR: 0.55, 95% CI: 0.32–0.94) but no OS difference (HR: 0.84, 95% CI: 0.50–1.40) [60•]. Tivozanib also showed a favorable toxicity profile with less diarrhea (any grade: 35% vs 57%; grade 3: 2% vs 9%), less hand foot syndrome (any grade: 17% vs 46%; grade 3: 1% vs 10%), and better tolerability (dose interruption due to treated-related adverse events: 48% vs 63%; dose reduction due to treated-related adverse events: 24% vs 38%).

## Rechallenge of ICI

With the rapid adoptions of ICI-based combinations in the frontline setting, a clinically relevant question was whether there would be a role for ICI rechallenge after progression of prior ICIs. Small datasets from retrospective studies suggested the ORR were 23–25% [61, 62]. Recently, several prospective phase II trials investigated an adaptive approach (OMNIVORE, TITAN-RCC, HCRN GU16-260): nivolumab monotherapy was initiated as frontline therapy with ipilimumab/nivolumab as the salvage treatment for non-responders or stable disease. The ORRs for salvage approach were generally not encouraging (4% (OMNIVORE), 16% (TITAN-RCC), 11.4% (HCRN GU16-260)) and did not support such response-adaptive strategy [44, 63, 64] (Table 5).

**Table 5:** Trials of ICI for refractory mRCC

	Study design	Histology	Treatment	Efficacy
OMNIVORE (Part B, *N* = 57)	Phase II (adaptive design)	ccRCC and nccRCC	Two doses of ipilimumab/nivolumab for non-responders to nivolumab monotherapy	ORR: 4% 90% CI: 1-11
TITAN-RCC (First-line subgroup, *N* = 65)	Phase II (adaptive design)	ccRCC (IMDC intermediate/poor risk)	2 or 4 doses of ipilimumab/nivolumab for non-responders to nivolumab monotherapy	ORR: 17% mPFS: 6.3 months 95% CI: 3.7-10.1 mOS: 27.2 months 95% CI: 19.9-NE
HCRN GU 16-260: Cohort A (Part B, *N* = 35)	Phase II (adaptive design)	ccRCC	4 doses of ipilimumab/nivolumab for non-responders to nivolumab monotherapy	ORR: 11.4%
Fraction-RCC (Track 2, *N* = 46)	Phase II (randomized platform trial)	ccRCC	4 doses of ipilimumab/nivolumab for ICI-treated ccRCC	ORR: 17.4% 95% CI: 7.8-31.4 mDOR: 16.4 months 95% CI: 2.1-27 mPFS: 3.7 months 95% CI: 2.0-7.3 mOS: 23.8 months 95% CI: 13.2-NE
KEYNOTE-146 (ICI-treated subgroup, *N* = 104)	Phase 1b/II (single arm)		Pembrolizumab/lenvatinib for ICI-treated	ORR: 56% 95% CI: 45.7-65.5 mDOR: 12.5 months 95% CI: 9.1-17.5) mPFS: 12.2 95% CI: 9.5-17.7 mOS: NR 95% CI: NR-NR
CONTACT-03 (*N* = 522)	Phase III (randomized study)		Atezolizumab/cabozantinib vs cabozantinib	mPFS: 10.6 vs 10.8 months HR: 1.03 95% CI: 0.83-1.28 mOS: 25.7 vs NE HR: 0.94 95% CI: 0.70-1.27 ORR: 41% vs 41%

Fraction-RCC is a signal-seeking randomized phase II trial which evaluated ICI combinations in advanced RCC who had previously progressed on ICI therapy (track 2) [65•]. For patients who were randomized to ipilimumab/nivolumab arm, eight patients (8/46) achieved partial response and zero patients had CR. The ORR was 17.4% (95% CI: 7.8–31.4). The median PFS was 3.7 months (95 CI: 2.0–7.3), and median OS was 23.8 months (95% CI: 13.2–not estimable). Of note, although the ORR was modest, the median DOR was 16.4 (95% CI: 2.1–27) suggesting a small subset of patients may derive benefit with ipilimumab/nivolumab after progression of prior PD-1/PD-L1 inhibitors, though data need to be interpreted with caution.

For ICI-TKI combination in the refractory setting, KEYNOTE-146 evaluated pembrolizumab/lenvatinib in a phase IB/II single-arm study [66•]. The ICI-treated subgroup required disease progression of prior anti-PD-1/PD-L1 regimens. After a median follow-up of 19.8 months, in the ICI-treated subgroup (*N* = 104; 65% had previous anti-VEGF therapies), the ORR was 55.8% (95 CI: 45.7–65.5) with a median DOR of 12.5 months (95% CI: 9.1–17.5). The median PFS was 12.2 months (95% CI: 9.5–17.7), and the OS was not reached. While these data are encouraging, it is difficult to isolate the effect of the component parts in this single arm study. The recently reported phase III CONTACT-03 study randomized ICI-treated mccRCC (*N* = 533) to atezolizumab (PD-L1 inhibitor)/cabozantinib vs cabozantinib [59••]. Patients who had disease progression during or after ICI (anti-PD-L1 or anti-PD-1) in the first-line or second-line setting and ICI being the immediately preceding line of therapy were eligible. The two-primary endpoints were PFS and OS. After a median follow-up of 15.2 months, there was neither a PFS (mPFS: 10.6 vs 10.8 months, HR: 1.03, 95% CI: 0.83–1.28) nor OS benefit (mOS: 25.7 vs NR months, HR: 0.94, 95% CI: 0.70–1.27) of atezolizumab/cabozantinib combination. More serious adverse events occurred in the atezolizumab/cabozantinib arm (48% vs 33%; adverse events leading to death: 6% vs 4%). The CONTACT-03 study did not support the PD-L1 inhibitor of atezolizumab/cabozantinib in previously ICI-treated mccRCC. A phase III randomized TiNiVO-2 study (NCT04987203) is on-going which investigates the PD-1 inhibitor of nivolumab in combination with tivozanib vs tivozanib monotherapy in ICI-treated mccRCC (recruitment goal: *N* = 326; primary endpoint: PFS) [67•].

## Role of cytoreductive nephrectomy

The OS benefit of upfront cytoreductive nephrectomy (CN) in mRCC was supported by two landmark trials conducted in the cytokine era [68, 69]. Although there had been no available prospective data evaluating CN in the early targeted therapy era, the benefit of CN was extrapolated from the two landmark trials and retrospective data suggested OS benefit of CN in the context of VEGF TKIs [70]. This practice was formally challenged after the results from the landmark CARMENA trial. CARMENA was a phase III, noninferiority trial that investigated sunitinib alone vs CN followed by sunitinib (CN-sunitinib) in 450 MSKCC intermediate and poor risk mRCC [71]. The non-inferiority margin was set as the upper boundary of the 95% CI of the death hazard ratio ≤1.20. The initial results supported noninferiority of sunitinib alone compared to CN-sunitinib (median OS: 18.4 vs. 13.9 months, HR 0.89, 95% CI: 0.71 to 1.10) [71]. However, the results also engendered criticism for disproportionate accrual of poor risk patients (44%) which left unanswered question for the true role of CN in selected intermediate risk patients. An updated analysis with longer follow-up continued to show noninferiority of sunitinib alone (HR: 0.97, 95% CI, 0.79–1.19) and numerically longer OS (19.8 vs 15.6 months) compared to CN-sunitinib in the ITT population. Patients were further reclassified with the IMDC criteria in the post hoc analysis. Among IMDC intermediate risk, sunitinib alone had numerically longer OS (27.9 vs 19 months) although the results did not meet noninferiority (HR: 0.94, 95% CI: 0.70–1.24). When stratified by the number of risk factors (1 or 2), the OS favored CN-nephrectomy among patients with only one IMDC risk factor (31.4 vs 25.2 months) but not among patients with two IMDC risk factors (17.6 vs 31.2 months). Post hoc analysis was also conducted by the number of metastatic sites (1 vs ≥ 2). The OS favored upfront CN over sunitinib alone for patients with only one metastasis (OS: 23.2 vs 22.7 months) but not for patients with ≥2 metastasis (OS: 14.4 vs 16.7 months). Taken together the above analyses, CARMENA emphasized the importance of thorough patient selection (e.g., IMDC risk 0 or 1 and low-volume disease) in identifying patients for upfront CN. Another phase III SURTIME trial randomized mccRCC patients to immediate CN vs 3 cycles of sunitinib followed by CN (deferred CN). Due to poor trial accrual, the final study included 99 patients and there was no progression-free benefit (primary endpoint) of immediate CN vs deferred CN. Patients in the deferred CN arm had longer OS (32.4 vs 15 months, HR: 0.57, 95% CI: 0.34–0.95).

Based on the results from CARMENA and SURTIME, the utilization of upfront CN has been declining as reflected in the recent landmark ICI-doublet trials. However, the timing and eligibility for CN are still unclear in the contemporary kidney cancer patients receiving frontline ICI regimens. There are currently several prospective clinical trials that are evaluating the role of CN in the immunotherapy era including PROBE [72] (NCT04510597), NORDIC-SUN [73] (NCT03977571), and Cyto-KIK [73] (NCT04322955).

## Role of radiation therapy

### SBRT for primary RCC

Historically, RCC was considered to be a radioresistant malignancy when utilized in the context of conventional fractionation. However, when human RCC cell lines were exposed to increased radiation doses, there are an exponential decrease in survival compared to the minimal survival effects from conventional radiation [74]. Stereotactic body radiation therapy (SBRT, dose ≥ 5 Gy delivered in five or fewer fractions) provides high-dose and precise conformal radiation, and its clinical efficacy in inoperable primary kidney cancer tumor has been reported since the early 2000s [75–77]. SBRT is an effective alternative and offers noninvasive cytoreduction to nonsurgical candidates while potentially preserving kidney function [78]. Additionally, SBRT has the potential to augment the anti-tumor immune response via increase in tumor-antigen presentation and immune-cell infiltration and therefore has the potential to increase the efficacy of ICI in mRCC [79–81]. This concept is currently being investigated in the phase II SAMURAI study which randomizes patients in a 2:1 ratio to receive SBRT (42 Gy in 3 fractions) plus ICI-doublet vs ICI-doublet alone (NCT05327686, NRG GU-012) in IMDC intermediate/poor risk patients [82•]. Another similar phase II study, CYTOSHRINK, randomizes patients (2:1 ratio) to SBRT (30–40 Gy in five fractions) plus ipilimumab/nivolumab vs ipilimumab/nivolumab in IMDC intermediate/poor RCC (NCT04090710) [83]. Both studies allow mRCC with any histology.

### Oligometastatic

Oligometastatic (OM) disease involves metastatic lesions with limited spread; this is typically a maximum of five lesions, though criteria to define oligometastatic disease across solid tumor malignancies are evolving [84]. Metastatic-directed therapy (MDT) was historically performed through surgical measures, but has more recently expanded to include SBRT [85]. Several studies have investigated SBRT for OM RCC [86]. A single-arm study by Tang et el. studied oligometastatic RCC (≤5 metastases; with no more than one line of prior systemic therapy; IMDC favorable risk: 47%, intermediate risk: 50%) to receive SBRT to all lesions and maintained off systemic therapy. The median PFS was 22.7 months (1-year PFS of 64% (95% CI: 48–85)), and the 1-year adjusted systemic therapy-free survival was 86% [87•]. In another single-arm study of SBRT by Hannan et al. was investigated in treatment naïve OM RCC with ≤3 extracranial metastases (74% IMDC favorable and 26% intermediate risk). The median time to start of systemic therapy (primary endpoint) was 26.6 months (interquartile range: 16.3–30.3), the 1-year freedom from systemic therapy probability was 91.3% (95% CI: 69.5–97.8), and the 1-year PFS was 82.6% (95% CI, 60.1–93.1) [88•]. Shiva et al. investigated SBRT in OM RCC (≤5 metastases) with ≤ two lines of prior systemic therapy followed by eight cycles of pembrolizumab (200 mg, every 3 weeks). The ORR was 63%, and disease control rate was 83%. The 12-month and 24-month PFS were 60% and 45%, respectively, and the OS was 90% and 74%, respectively [89].

### Oligoprogression

Oligoprogression (OP) in mRCC typically means an individual who has disease progression of a select number of metastatic lesions, while other metastatic sites remain responsive/stable to a given systemic therapy. SBRT to those progression sites could achieve desirable disease control while extending the duration of the given systemic agent [86]. A phase II single-arm study investigated SBRT in IMDC favorable/intermediate mRCC who had OP after ≥ 3 months of TKI therapy. This trial closed prematurely due to slow accrual after 38 patients were enrolled. The median PFS was 9.3 months (95% CI: 7.5–15.7), and the median time to change systemic therapy was 12.6 months (95% CI: 9.6–17.4). Another phase II single-arm study (*N* = 20) reported SBRT delayed new systemic therapy by >6 months in 70% patients with OP mRCC (median: 11 months, 95% CI: 4.5–19.3). Of note, patients on ICI in this trial had longer PFS compared to TKI, suggesting a synergistic effect of IO and SBRT combination [88•]. Ongoing prospective trials are investigating ICI and SBRT combination in OP RCC (NCT04974671, NCT04299646).

## Special considerations for non-clear cell RCC

nccRCC represents a biologically heterogeneous disease entity and roughly 20–25% of the kidney cancer belongs to nccRCC, with papillary RCC being the most common (10–15%), followed by chromophobe RCC (5%). Other subtypes such as medullary RCC, collecting duct RCC, and unclassified RCC represent <1% of nccRCC. Of note, the WHO published the fifth edition classification of urogenital tumors in 2022. This version did not classify papillary RCC into type 1 or type 2. Additionally, it added a molecular-defined RCC category (e.g., fumarate hydratase-deficient RCC, succinate dehydrogenase-deficient RCC, SMARCB1-deficient RCC, ALK-rearranged RCC) in addition to morphology-based classification. Given the scarcity of nccRCC, it is challenging to conduct large trials in this disease space. The current available systemic agents such as TKIs and ICIs have various activities in nccRCC, but the efficacy is generally lower compared to ccRCC. In addition, most of the data is driven by papillary RCC with mixed representation of other subtypes in the datasets [90].

MET proto-oncogene alterations are commonly found in papillary RCC [91]. Cabozantinib, the multi-targeted (MET/VEGF/AXL/RET/KIT) TKI, has the most robust activity as a single agent in this histology. The phase II randomized PAMPET study investigated cabozantinib vs sunitinib in the papillary RCC which demonstrated longer PFS (median: 9 vs 5.6 months, HR: 0.60, 95% CI: 0.37–0.97) and higher ORR (23% vs 4%). For ICI monotherapy, the ORR of nivolumab in nccRCC was around 13–14% in two single-arm studies (CheckMate-374 cohort B, *N* = 44 (55% was papillary RCC); HCRN GU 16-260 cohort B (part A), *N* = 35 (54% was papillary RCC)) [92, 93]. Pembrolizumab had an ORR of 27% in the single-arm KEYNOTE-427 (cohort B, *N* = 165 (72% was papillary RCC)) with a median DOR of 29 months [94•]. In the papillary histology, the ORR was 29% (95% CI: 21–38%). With regard to the ICI-based doublets, ipilimumab/nivolumab was reported to have an ORR of 20% (95% CI: 9–34%) in the CheckMate 920 (*N* = 52; 42% was unclassified and 35% was papillary) [95]. Atezolizumab/bevacizumab was investigated in a phase II study of advanced RCC. Among the subgroup of nccRCC (*N* = 42, papillary: 35%, chromophobe: 29%; unclassified: 26%), the ORR was 26% [96]. Nivolumab/cabozantinib was investigated in a single-center study which included two cohorts of nccRCC [97]. Cohort A (*N* = 40 patients; papillary: 80%, unclassified: 15%, translocation-associated: 5%) reported an ORR of 48% (95% CI: 32–64%); cohort B included seven chromophobe patients, and none of them had response. This cohort was closed early for futility. Pembrolizumab/lenvatinib was investigated in a multi-center single-arm study (KEYNOTE-B61) in nccRCC (*N* = 158; papillary: 58%, chromophobe: 18%, unclassified: 13%) [98•]. The ORR was 49% (95% CI: 41–57) in all nccRCC and 54% (95% CI: 43-64) in papillary RCC including a CR rate of 9%. For chromophobe (*N* = 29), eight patients achieved partial response (ORR: 28%, 95% CI:13–47).

In summary, aside from papillary RCC, the contemporary ICI-based regimens have modest activity in other subtypes of nccRCC. In addition, evidence to support subsequent lines of therapy is elusive. Future prospective studies targeting novel mechanisms in nccRCC are warranted to fill the unmet needs.

## Conclusions

With the advent of new treatment modalities including ablative techniques, SBRT, and ICIs, the OS of patients with RCC have prolonged significantly across the disease spectrum. ICIs have brought the possibilities of cure even in advanced disease. With improved understanding of the RCC biology and cancer immunology, the development of novel therapeutics is anticipated in near future. The knowledge of ideal treatment sequencing will continue to evolve.
